# Paraneoplastic Anti-transcriptional Intermediary Factor 1γ (Anti-TIF1γ)-Positive Dermatomyositis Presenting as Dropped Head Syndrome: A Case Report

**DOI:** 10.7759/cureus.79631

**Published:** 2025-02-25

**Authors:** Areti Kalfoutzou, Christos Toilos, Cleopatra Rapti, Dimitra Manoli, Evangelos Kouremenos, Nikolaos Chaleplidis, Asimina Restemi, Eleftheria Bagiokou, Vasileios Ramfidis

**Affiliations:** 1 Department of Medical Oncology, 251 Air Force General Hospital, Athens, GRC; 2 Department of Neurology, 251 Air Force General Hospital, Athens, GRC; 3 Department of Pathology, 251 Air Force General Hospital, Athens, GRC

**Keywords:** autoantibodies, dermatomyositis, dropped head syndrome, paraneoplastic syndrome, squamous cell carcinoma

## Abstract

Anti-transcriptional intermediary factor 1γ (anti-TIF1γ) antibody-positive dermatomyositis is a rare but significant subtype of dermatomyositis, often associated with malignancy. We present a case of a 66-year-old male with a history of heavy smoking, who was diagnosed with a locally advanced squamous cell carcinoma of the right cervical lymph nodes. After treatment with chemoradiotherapy, he developed dropped head syndrome and muscle weakness and was found to have elevated anti-TIF1γ antibodies, suggestive of paraneoplastic myositis. Despite aggressive treatment, the patient’s condition deteriorated, leading to recurrent carcinoma and, ultimately, death due to respiratory infection. The rarity of this presentation and the diagnostic challenges are discussed, emphasizing the role of TIF1γ antibodies in diagnosing this rare entity.

## Introduction

Dermatomyositis is an autoimmune inflammatory myopathy frequently seen in cancer patients, particularly as part of a paraneoplastic syndrome [1]. It is characterized by the presence of specific autoantibodies, which define its clinical profile [2]. Anti-transcriptional intermediary factor 1γ (anti-TIF1γ) antibody-positive dermatomyositis is a specific subtype of dermatomyositis with an established link to several malignancies, with up to half of adult patients harboring an underlying or concurrent cancer [2]. Thus, the presence of anti-TIF1γ autoantibodies warrants a vigilant oncologic workup and close follow-up for early detection of malignancy or cancer recurrence [3]. Nevertheless, the clinical spectrum of dermatomyositis extends beyond its classic presentation, with atypical muscle and cutaneous manifestations that can significantly delay diagnosis. Our case aims to shed light on the rare clinical manifestation of dropped head syndrome as an initial sign of anti-TIF1γ-positive dermatomyositis, emphasizing early recognition and comprehensive evaluation of paraneoplastic syndromes in cancer patients.

## Case presentation

**Figure 1:**
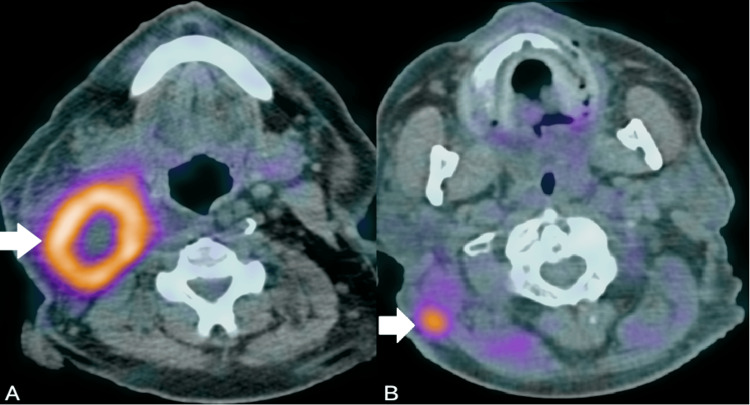
FDG PET scan of the patient at initial presentation. FDG PET scan of the patient at initial presentation demonstrating a hypermetabolic right cervical lymph node block, suspicious for malignancy (white arrow - panel A). FDG PET scan of the patient at recurrence, displaying a hypermetabolic right sternocleidomastoid lesion, suspicious of cancer recurrence (white arrow - panel B). FDG PET: Fluorodeoxyglucose Positron Emission Tomography

A 66-year-old male with a history of long-term smoking (60 packs/year), hyperuricemia, hyperlipidemia, and arterial hypertension presented in January 2022 with self-detected enlarged lymph nodes in the right submandibular region for the past two months. Nasal and pharyngeal endoscopy was insignificant. A fluorodeoxyglucose positron emission tomography (FDG PET) scan revealed multiple hypermetabolic lymph nodes in the right cervical area (Standard Uptake Value, or SUVmax: 17.2) (Figure 1A). A core biopsy of the cervical lymph node was performed, and histopathology was consistent with squamous cell carcinoma, human papillomavirus (HPV)-related. The patient underwent an extended right cervical lymph node dissection, and the surgical specimen histopathology confirmed the presence of grade III squamous cell carcinoma, with 15 out of 30 resected lymph nodes infiltrated (stage N3b, American Joint Committee on Cancer (AJCC) 8th edition). The resection status was R0, with a margin distance of 0.4 cm, and p16 staining was negative on the tumor cells.

The patient underwent a total of 30 sessions of image-guided radiotherapy (IGRT). These sessions targeted the tumor bed (total dose: 64.5 Gy), as well as the non-infiltrated left cervical lymph nodes (total dose: 50.5 Gy), and encompassed the nasopharynx, pharynx, base of the tongue, and supraclavicular lymph nodes (total dose: 54 Gy). This radiotherapy was conducted concurrently with radiosensitizing chemotherapy, consisting of seven cycles of weekly cisplatin at a dose of 40 mg/m². Two months post-completion of chemoradiotherapy, a follow-up computed tomography (CT) scan of the chest revealed a pulmonary embolism in the major branch of the right pulmonary artery, which was managed with antithrombotic socks and tinzaparin at a dosage of 14,000 IU for six months.

Ten months after completing chemoradiotherapy, the patient presented at a follow-up appointment with an inability to raise his head, a symptom that had persisted for the past month. Furthermore, he reported dysphagia and loss of appetite for the past two weeks. Upon clinical examination, the patient exhibited erythema on the face and neck in a V-neck pattern. A neurological examination indicated dropped head syndrome, a right-winged scapula, and, more specifically, weakness of the head extensor muscles (muscle strength rated as 2/5), the right sternocleidomastoid muscle (muscle strength 3/5), and the right upper limb with a C5-C6 nerve distribution (muscle strength 4/5), without fluctuation. No other focal neurological deficits were observed.

Based on the clinical presentation, we initially proceeded with neurophysiology testing. Nerve conduction studies revealed evidence of asymmetric sensorimotor mixed polyneuropathy, which can be attributed to previous chemotherapy, whereas needle electromyography (EMG) showed motor unit action potentials of short duration, low amplitude, and increased polyphasia in the cervical paraspinal muscles, the right upper part of the trapezius muscle, bilateral deltoid muscles, and right infraspinatus muscle, as well as spontaneous activity, fibrillation potentials, and positive sharp waves in the right infraspinatus muscle, the right upper part of the trapezius muscle, and the right sternocleidomastoid muscle. No myokymic discharges were detected. These EMG findings were indicative of a myopathic process. Repetitive nerve stimulation studies were negative for neuromuscular junction disorders.

Standard laboratory examinations were within normal limits, apart from a low thyroid-stimulating hormone (TSH: 12.26 μIU/mL) and free thyroxine (FT4: 0.25 ng/dL) (Table 1). Immunologic assay revealed the presence of anti-nuclear antibodies (ANAs) with a positive titer of 1:640 (Table 1). Serum anti-muscle-specific kinase (anti-MuSK) and anti-acetylcholine receptor (anti-AChR) antibodies yielded negative results (Table 2). Additionally, a lumbar puncture (LP) was performed. The cerebrospinal fluid (CSF) analysis revealed no cells, a protein level of 36.5 mg/dL, and a glucose level of 59 mg/dL, whereas oligoclonal bands were not identified. In the context of investigating a possible paraneoplastic neurological disorder and the indicative myopathic process in the electroneuromyography (ENMG), serum paraneoplastic antibody testing revealed elevated TIF1γ antibodies (91 U/mL), weakly positive Ro52 antibodies (14 U/mL), and indeterminate Ku antibody levels (8 U/mL) (Table 2).

**Table 1:** Laboratory examinations of the patient upon admission.

Type of examination	Patient’s values	Normal range
White blood cell (WBC) count	4.2	4-10 × 10⁹/L
Neutrophils	2.4	1.5-7 × 10⁹/L
Hemoglobin	127	140-180 g/L
Hematocrit	38.9	42-52%
Platelets	367	140-440 × 10⁹/L
Blood urea nitrogen (BUN)	11.07	5.35-19.27 mmol/L
C-reactive protein (CRP)	13.98	0-10 mg/L
Erythrocyte sedimentation rate (ESR)	16	0-20 mm/h
Lactate dehydrogenase (LDH)	299	100-230 IU/L
Creatinine	0.8	0.7-1.3 mg/dL
Creatinine kinase (CK)	117	10-195 IU/L
Myoglobin	90	10-92 ng/mL
Parathormone (PTH)	46	10-65 pg/mL
Free thyroxine (FT4)	0.25	0.89-1.76 ng/dL
Thyroid-stimulating hormone (TSH)	12.26	0.35-4.94 μIU/mL
Adrenocorticotropic hormone (ACTH)	24	10-60 pg/mL
Anti-nuclear antibodies (ANAs)	1:640	1:40

**Table 2:** Serum analysis for paraneoplastic antibodies. Normal ranges: negative (0-5), indeterminate (6-10), weakly positive (11-25), positive (26-50), strongly positive (51-150). For Anti-AChR, normal ranges: negative (0-0.15 nM), indeterminate (0.2-0.5 nM), positive (≥0.6). Mi-2a: Chromodomain Helicase DNA-binding Protein 4a; Mi-2b: Chromodomain Helicase DNA-binding Protein 4b; Ku: Ku Antigen; PM-Scl100: Polymyositis-Scleroderma 100 kDa; PM-Scl75: Polymyositis-Scleroderma 75 kDa; SRP: Signal Recognition Particle; Jo-1: Histidyl-tRNA Synthetase; PL-7: Threonyl-tRNA Synthetase; PL-12: Alanyl-tRNA Synthetase; OJ: Isoleucyl-tRNA Synthetase; EJ: Glycyl-tRNA Synthetase; Ro-52: SSA/Ro 52-kDa Protein; TIF1γ: Transcription Intermediary Factor 1 Gamma; MDA5: Melanoma Differentiation-Associated Protein 5; NXP2: Nuclear Matrix Protein 2; SAE1: Small Ubiquitin-like Modifier Activating Enzyme 1; Anti-AChR: Anti-Acetylcholine Receptor; Anti-MuSK: Anti-Muscle-Specific Kinase

Antibody type	Result
Mi-2a	Negative
Mi-2b	Negative
Ku	Indeterminate (8)
PM-Scl100	Negative
PM-Scl.75	Negative
SRP	Negative
Jo-1	Negative
PL-7	Negative
PI-12	Negative
OJ	Negative
EJ	Negative
Ro-52	Weakly positive (14)
TIF1γ	Strongly positive (91)
MDA5	Negative
NXP2	Negative
SAE1	Negative
Anti-AChR	Negative
Anti-MuSK	Negative

A magnetic resonance imaging (MRI) scan of the brain with intravenous contrast revealed changes consistent with microischemic leukoencephalopathy. An MRI scan of the cervical spine, right brachial plexus, and scapula showed significant atrophy of the right sternocleidomastoid muscle, as well as changes consistent with the patient’s history of surgery and subsequent radiotherapy. Due to concerns about recurrent disease, the patient underwent an FDG PET scan, demonstrating significant hypermetabolic activity in the subcutaneous tissue of the right upper lateral cervical area, extending to the posterior border of the right sternocleidomastoid muscle (SUVmax: 7.5) (Figure 1B). Furthermore, there were multiple hypermetabolic subpleural nodules at the base of the right hemithorax (SUVmax: 9.5), mediastinal lymph nodes, mainly in the paratracheal region (SUVmax: 8), and a lesion in the O1 vertebra (SUVmax: 14.1).

**Figure 2:**
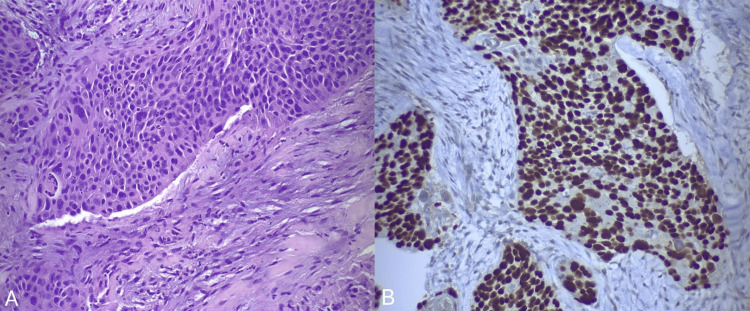
Sternocleidomastoid lesion biopsy specimen, hematoxylin-eosin stain, x20 magnification: moderately to poorly differentiated squamous cell carcinoma. This micrograph demonstrates significant nuclear pleomorphism. Keratinization is inconspicuous, suggesting a lower level of differentiation. The tumor cells are embedded within a desmoplastic stroma, indicative of an intense stromal reaction (panel A). Immunohistochemical analysis shows diffusely positive staining for p40, a nuclear marker specific to squamous cell carcinoma (panel B).

**Figure 3:**
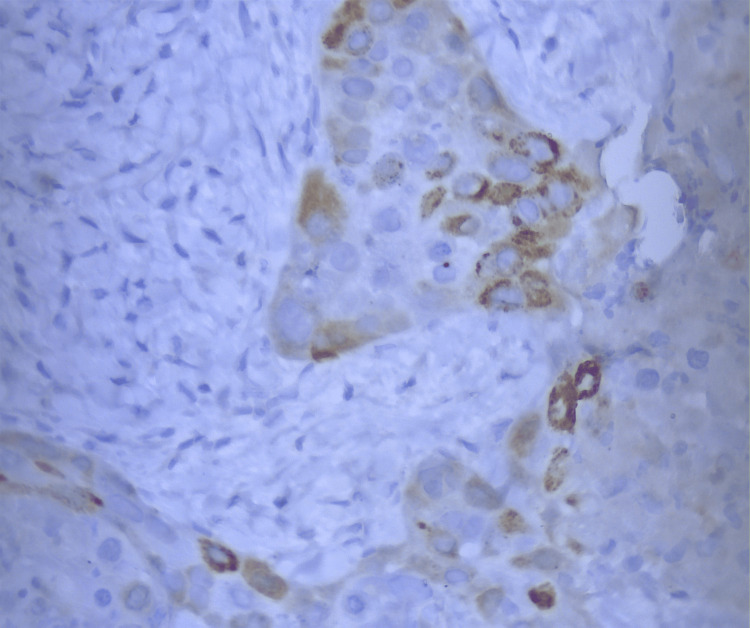
Immunohistochemical analysis of the cervical lymph node specimen. Immunohistochemical analysis of the cervical lymph node specimen demonstrates focal positive staining for p16, observed in both nuclear and cytoplasmic regions of cells. The sporadic nature of the staining indicates a heterogeneous expression pattern of p16 within the examined tissue sample (×40 magnification).

Percutaneous imaging-guided biopsy of a suspicious lung nodule and the hypermetabolic sternocleidomastoid lesion confirmed the recurrence of the patient’s squamous cell carcinoma. Histopathological examination of both specimens revealed basophilic small to medium-sized cells with focal necrosis, embedded within a desmoplastic stroma (Figure 2A). Immunohistochemical analysis of the cervical lesion specimen showed positive staining for p40 (Figure 2B) and p53, focal positive staining for p16 (Figure 3), and a Ki67 proliferation index of 50%-60%. In contrast, the immunohistochemical examination of the lung nodule specimen demonstrated positive staining for p63 and negative staining for p16. These findings are strongly suggestive of paraneoplastic dermatomyositis with positive serum anti-TIF1γ antibodies.

The case was discussed in a Multidisciplinary Tumor Board (MDT), and the initiation of systemic treatment with a combination of carboplatin, 5-fluorouracil (5-FU), and pembrolizumab was decided. Additionally, the patient was prescribed methylprednisolone 24 mg per dose daily for a month, followed by a gradual tapering. After completion of corticosteroid treatment and the first chemotherapy cycle, significant improvement in weakness of the head extensor muscles was observed, allowing the patient to hold his head up independently. Moreover, the patient experienced a noticeable improvement in dysphagia, with increased ease in swallowing. Additionally, the patient underwent radiotherapy for the lumbar spine, targeting a painful bone-lytic lesion in the O1 vertebra. After completing two cycles of therapy, the patient was admitted to the hospital due to a high-grade fever (t: 39.2°C) and productive cough. Treatment with broad-spectrum antibiotics, including coverage for *Pneumocystis carinii* (PCP), along with high doses of prednisolone (1 mg/kg), was initiated. Repeated sputum and blood cultures, as well as fungal investigations and COVID testing, yielded negative results. A CT scan of the chest revealed bilateral interstitial infiltrates in the lower lobes. A bronchoalveolar lavage (BAL) was advised but was ultimately not feasible due to the patient’s rapidly deteriorating condition, ultimately leading to his death despite intensive management.

## Discussion

Dermatomyositis describes a wide spectrum of autoimmune myopathies that manifest with chronic inflammation and/or skin disorders [1]. These entities are often characterized by the production of specific autoantibodies targeting nuclear or cytoplasmic antigens, known as myositis-specific autoantibodies, including anti-Mi-2 helicase (anti-Mi-2), anti-small ubiquitin-like modifier activating enzyme (anti-SAE), anti-melanoma differentiation-associated gene 5 (anti-MDA5), autoantibodies against aminoacyl-tRNA synthetases (anti-ARS), anti-nuclear matrix protein 2 (anti-NXP2), anti-TIF1γ, anti-histidyl-tRNA synthetase (anti-Jo-1) antibodies, among others [1]. Anti-TIF1γ-positive dermatomyositis is a unique subtype marked by the presence of antibodies against TIF1γ in the serum, a 155-kDa nuclear protein that acts as a tumor suppressor gene and is implicated in multiple mechanisms of DNA transcription and repair through the regulation of the Smad/transforming growth factor-beta (TGF-β) and Wnt/β-catenin pathways [2-4]. Its incidence is higher in adults over 60, exhibiting a male predominance [5].

The link between anti-TIF1γ-positive dermatomyositis and cancer is well-established. About 30% of dermatomyositis cases in adults are associated with malignancy, whereas up to 50%-75% of dermatomyositis cases in cancer patients are found to have positive serum anti-TIF1γ antibodies [6]. Anti-TIF1γ-positive dermatomyositis is encountered in several tumor types, including lung, breast, ovarian, colorectal, esophagogastric, squamous, and bladder cancer [7-9].

Clinical presentation consists of symptoms involving mainly the skeletal muscles and skin. Patients most commonly present with symmetrical proximal muscle weakness, along with dysphagia [7]. Dropped head syndrome, characterized by severe weakness of the neck extensor muscles (or increased tone of flexor muscles), with progressively induced kyphosis and inability to lift the head, is a rare clinical manifestation of inflammatory myopathies, including dermatomyositis [10]. Typical skin manifestations include V-neck signs, heliotrope rash, Gottron’s papules, diffuse photodermatitis, and hyperkeratotic papules on palms, whereas Raynaud’s syndrome is more uncommon [3,11].

Laboratory examinations usually reveal elevated creatine kinase (CK), myoglobin, or lactate dehydrogenase (LDH), and inflammatory markers such as C-reactive protein (CRP) and erythrocyte sedimentation rate (ESR) [9,12]. The presence of serum myositis-specific antibodies confirms the diagnosis and classifies the subtype of dermatomyositis. Anti-TIF1γ antibodies have a reported sensitivity of 78% and specificity of 89% for the diagnosis of anti-TIF1γ-positive dermatomyositis [9]. Additionally, histopathological examination from a skin or muscle biopsy may reveal perivascular infiltrates composed of lymphocytes, with scattered neutrophils and eosinophils, along with myocyte degeneration and regeneration [13,14]. Our case did not demonstrate laboratory signs of muscle destruction, whereas hypothyroidism was attributed to previous neck irradiation.

Due to a high probability of malignancy in patients with positive serum anti-TIF1γ antibodies, an extensive workup for malignancy should be carried out after diagnosis of anti-TIF1γ-positive dermatomyositis [9,15]. In the absence of specific symptoms, age- and gender-specific tests, such as mammography, transabdominal or transvaginal ultrasound, and imaging studies such as CT scans, MRI scans, or FDG PET scans, are capable of localizing the underlying malignancy [9]. In cases with positive serum anti-TIF1γ antibodies, where an extensive workup does not reveal an underlying malignancy, close follow-up and cancer screening for at least three to five years is strongly recommended [12].

The differential diagnosis of a patient presenting with dropped head syndrome includes neuromuscular junction disorders, such as myasthenia gravis, and myopathies, which can be distinguished into inflammatory myopathies such as poly/dermatomyositis, non-inflammatory myopathies such as isolated neck extensor myopathy (INEM) and post-radiation myopathy, and myopathies due to endocrine or metabolic disorders, or even systemic diseases such as amyloidosis [16,17]. Furthermore, neurological disorders, such as amyotrophic lateral sclerosis, Parkinson’s disease, and chronic inflammatory demyelinating polyneuropathy, should not be omitted from the differential diagnosis of dropped head syndrome (Table 3) [18].

**Table 3:** Conditions associated with dropped head syndrome. Table credit: [18] PM/Scl: Polymyositis/Scleroderma; GAD: Glutamic Acid Decarboxylase; MEK: Mitogen-Activated Protein Kinase

Category	Conditions
Neuromuscular junction disorders	Myasthenia gravis, Lambert-Eaton syndrome
Inflammatory myopathies	Polymyositis/Dermatomyositis, Scleromyositis (Overlap Syndrome - anti-PM/Scl), Isolated inflammatory axial myopathy, Anti-GAD associated inflammatory myopathy, Inclusion body myositis
Primary, non-inflammatory myopathies	Isolated neck extensor myopathy, Hereditary inclusion body myopathy, Mitochondrial myopathy, Nemaline myopathy (Adult onset), Facioscapulohumeral muscular dystrophy
Secondary myopathies	Post-radiation neck extensor myopathy, Post-botulinum toxin injection, Cushing syndrome, Carnitine deficiency, Hypothyroidism, Hypokalemia, Hyperparathyroidism
Neurological disorders	Amyotrophic lateral sclerosis, Parkinson’s disease, Multiple system atrophy, Cervical dystonia, Post-polio syndrome, Cervical myelopathy, Chronic inflammatory polyneuropathy, Tardive dyskinesia
Other causes	Malignancy, Post-surgical, Traumatic injury, Ankylosing spondylitis, Drug-induced (e.g., MEK inhibitors)

Considering the patient’s history and clinical and laboratory examinations, differential diagnoses in our case included hypothyroidism, post-radiation neck extensor myopathy, and paraneoplastic dermatomyositis [17]. Our patient received a cumulative dose of >50 Gy of radiation to the neck extensor muscles, which is known to potentially lead to post-radiation myopathy [17]. However, this diagnosis was considered less likely due to the presence of skin manifestations and the positive serum anti-TIF1γ antibodies. Additionally, dropped head syndrome has often been reported as a late-onset complication, occurring several years following radiotherapy, while our patient presented symptoms just a few months post-radiation [19].

The cornerstones of treatment for anti-TIF1γ-positive dermatomyositis involve addressing the underlying malignancy and administering corticosteroids or immunosuppressive therapy for the myositis [14]. Our patient received a combination of carboplatin, 5-FU, pembrolizumab, and corticosteroids. Despite these interventions, which led to an initial clinical improvement, his condition ultimately progressed, leading to disease progression and a fatal respiratory infection.

The prognosis of paraneoplastic dermatomyositis is poor, particularly due to the associated malignancy [15]. Most cancers are diagnosed within 12 months after the detection of serum anti-TIF1γ antibodies, while in some instances, dermatomyositis and cancer are simultaneously diagnosed [15]. During cancer treatment, serum TIF1γ antibodies have been proposed as a surrogate marker of disease progression and response to therapy [1].

To our knowledge, this is the first reported case of anti-TIF1γ-positive dermatomyositis presenting as dropped head syndrome, particularly as the initial clinical manifestation of the progression of the disease. This symptom, along with the patient’s dysphagia and V-neck erythema, as well as his previous cancer history, all pointed towards paraneoplastic dermatomyositis. Most reported cases of paraneoplastic myositis typically present with proximal muscle weakness, often accompanied by classic skin manifestations such as heliotrope rash, V-neck sign, and Gottron’s papules, whereas dropped head syndrome remains an extremely rare clinical presentation [1,3]. In our case, the presence of serum anti-TIF1γ antibodies confirmed the diagnosis of anti-TIF1γ-positive dermatomyositis, and a subsequent FDG PET scan detected the cancer recurrence.

## Conclusions

Anti-TIF1γ-positive dermatomyositis is a specific subtype of autoimmune myopathy, frequently presenting as a paraneoplastic syndrome in cancer patients. The presence of TIF1γ antibodies is a crucial diagnostic marker for its diagnosis. Despite aggressive treatment, its presence usually confers a poor prognosis on cancer patients. Early recognition and a multidisciplinary approach involving oncologists, pathologists, and neurologists are crucial steps in the early diagnosis of occult malignancy in patients with positive serum anti-TIF1γ antibodies, as well as in preventing long-term neurologic sequelae.
